# Machine learning-based prediction of diabetic peripheral neuropathy: model development and clinical validation

**DOI:** 10.3389/fendo.2025.1614657

**Published:** 2025-06-05

**Authors:** Meng Sun, Xingling Sun, Fei Wang, Li Liu

**Affiliations:** ^1^ Department of Neurosurgery, The First Affiliated Hospital of Shandong First Medical University and Shandong Provincial Qianfoshan Hospital, Jinan, Shandong, China; ^2^ Department of Nursing, The First Affiliated Hospital of Shandong First Medical University and Shandong Provincial Qianfoshan Hospital, Jinan, Shandong, China

**Keywords:** diabetic peripheral neuropathy, machine learning, interpretable, clinical data, risk prediction model

## Abstract

**Background:**

Diabetic peripheral neuropathy (DPN) is a common and debilitating complication of type 2 diabetes mellitus (T2DM), significantly impacting patients’ quality of life and increasing healthcare burdens. Early prediction and intervention are critical to mitigating its impact.

**Methods:**

This study analyzed 1,544 diabetic patients from the First Affiliated Hospital of Shandong First Medical University, who were randomly divided into a training cohort (n = 1,082) and a testing cohort (n = 462) using a 7:3 split ratio. Feature selection was performed using both Boruta and LASSO algorithms, and the intersection of the selected variables was used as the final predictor set. Eight key predictors were identified from 23 variables, including diabetes duration, uric acid, HbA1c, NLR, smoking status, SCR, LDH, and hypertension. Nine machine learning models were developed and compared for DPN risk prediction.

**Results:**

Stochastic Gradient Boosting (SGBT) demonstrated the best performance (training AUC: 0.933, 95% CI: 0.921–0.946; testing AUC: 0.811, 95% CI: 0.776–0.843). Shapley Additive Explanations (SHAP) analysis provided interpretability, highlighting the clinical importance of diabetes duration and HbA1c among other predictors.

**Conclusion:**

This study establishes a robust predictive tool for early DPN detection, laying the foundation for improved prevention and management strategies.

## 1 Introduction

The global prevalence of diabetes is rising at an unprecedented rate. In 2021, approximately 537 million adults (20–79 years) worldwide were living with diabetes, and this number is expected to increase to 783 million by 2045 (1). Diabetic peripheral neuropathy (DPN) is a common microvascular complication of type 2 diabetes (2–4), which significantly increases the risk of diabetic foot ulcers, non-traumatic lower limb amputations, and other related complications such as falls (5, 6). Furthermore, diabetic patients with peripheral neuropathy (PN) and foot ulcers have a more than two-fold increased relative risk of mortality compared to those without these two conditions (7). PN has been recognized as an independent risk factor for mortality in adults with diabetes (8). Therefore, there is an urgent need to develop novel strategies for the prevention and early diagnosis of DPN, which could help reduce associated disability and mortality rates, ultimately improving patient quality of life.

Machine learning (ML) technology has gained significant traction in the healthcare sector, demonstrating its potential in addressing complex medical challenges, including disease prediction and management (9–11). By leveraging large datasets, ML algorithms can uncover subtle patterns and relationships that are difficult to discern through traditional statistical methods.

In recent years, several studies have developed ML-based models to predict DPN, providing valuable insights into the feasibility of applying ML to this condition (12, 13). However, these studies often have limited clinical utility, as they typically rely on a single ML algorithm, lack model interpretability tools such as SHAP to explain feature contributions, and are rarely deployed in user-friendly formats like Shiny applications for real-world use. To overcome these limitations, we propose a comprehensive modeling strategy that incorporates robust feature selection (using Boruta and LASSO), systematic comparison of multiple machine learning algorithms to identify the best performer, and model interpretation through SHAP (Shapley Additive Explanations) to enhance transparency and clinical interpretability. We further proposed the development of a web-based risk calculator to enhance clinical implementation and support decision-making at the point of care. Given the substantial burden of DPN on affected individuals, the development of interpretable and accessible prediction tools has the potential to enable earlier diagnosis, guide preventive strategies, and improve patient outcomes.

This study aims to develop and validate an ML-based risk prediction model for DPN, using a combination of demographic, clinical, and biochemical parameters. By integrating robust ML algorithms and real-world clinical data, this research seeks to provide a valuable tool for clinicians to identify high-risk patients, enabling timely interventions and personalized treatment strategies to mitigate the impact of DPN.

## 2 Material and methods

### 2.1 Study population

**Figure 1:**
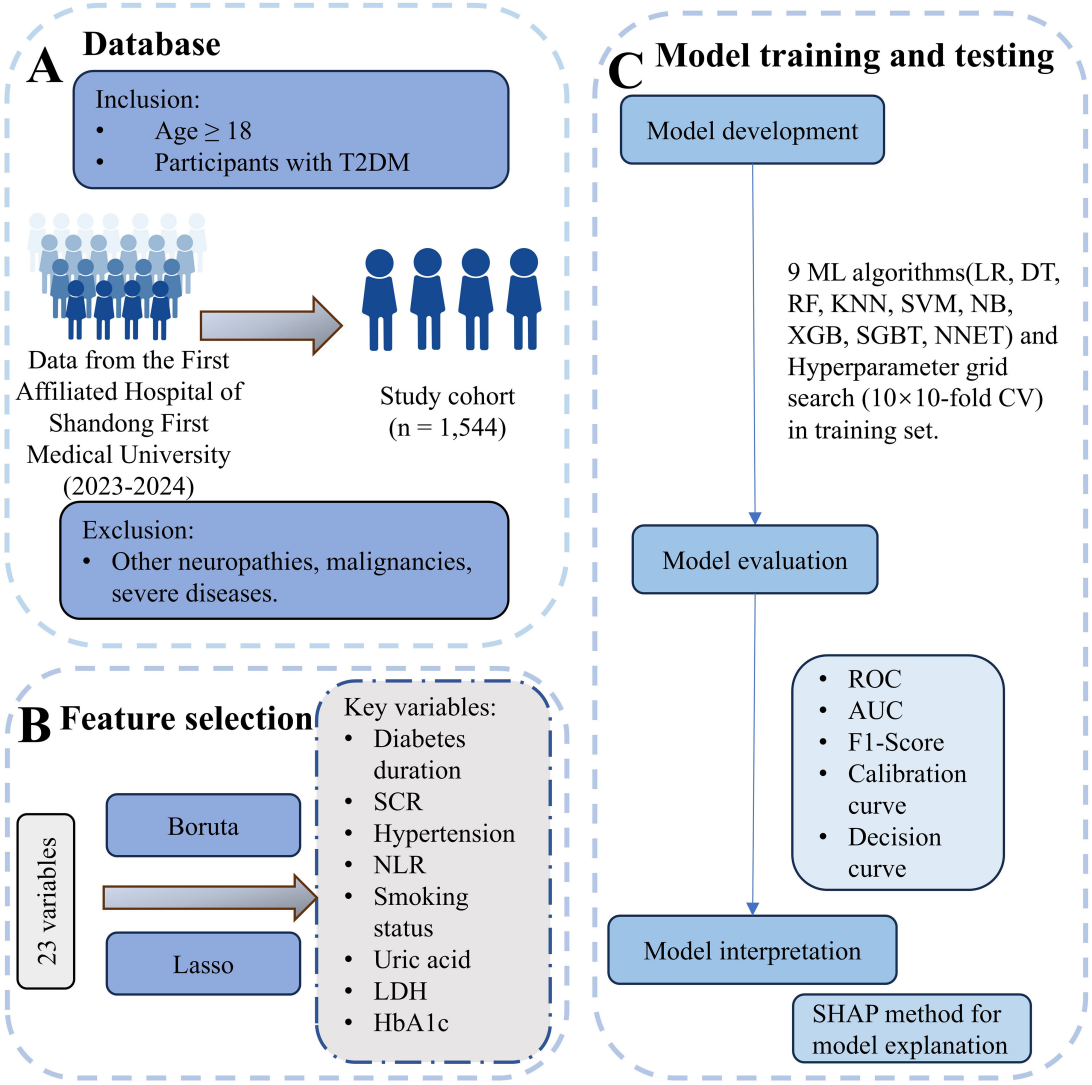
Overview of data processing and machine learning workflow. **(A)** Database: Participants were selected from The First Affiliated Hospital of Shandong First Medical University (2023–2024). Inclusion criteria were age ≥18 years and T2DM diagnosis. Exclusion criteria included other neuropathies, malignancies, severe infections, organ dysfunction, and metabolic disorders. The final study cohort comprised 1,544 individuals. **(B)** Feature selection: From an initial set of 23 variables, key variables were identified using the Boruta and LASSO methods. Selected features included diabetes duration, serum creatinine (SCR), hypertension, neutrophil-to-lymphocyte ratio (NLR), smoking status, uric acid, lactate dehydrogenase (LDH), and HbA1c. **(C)** Model training and testing: Nine machine learning algorithms, including Logistic Regression (LR), Decision Tree (DT), Random Forest (RF), K-Nearest Neighbors (KNN), Support Vector Machine (SVM), Naive Bayes (NB), XGBoost (XGB), Stochastic Gradient Boosting Trees (SGBT), and Neural Network (NNET), were applied, with hyperparameter optimization performed using 10×10-fold cross-validation. Model evaluation metrics included Receiver Operating Characteristic (ROC), Area Under the Curve (AUC), F1-score, calibration curves, and decision curves. Model interpretation was conducted using SHAP analysis for feature importance.

We collected data from 1,544 patients with type 2 diabetes mellitus (T2DM) who received treatment at the First Affiliated Hospital of Shandong First Medical University between January 2023 and December 2024. The inclusion criteria were: (1) age ≥18 years and (2) a diagnosis of T2DM. Exclusion criteria included other causes of peripheral neuropathy, malignant tumors, acute infectious diseases, severe hepatic or renal dysfunction, cardiac failure, metabolic disorders (such as thyroid disorders or vitamin B12 deficiency), and other severe life-threatening conditions. All patients underwent neurological assessments and nerve conduction studies (NCSs). DPN was diagnosed based on the presence of neuropathy-related clinical signs or symptoms and abnormal electromyography results, following the Toronto Expert Consensus (14). These participants were then randomly divided into training and testing groups in a 7:3 ratio. For participants with missing data, multiple imputations were performed using the “mice” package (n = 5). All variables had less than 30% missingness. Predictive mean matching was used for continuous variables, and logistic regression was applied for binary variables. Further details are provided in **Figure 1**. The study followed the principles of the Declaration of Helsinki and was approved by the Ethics Committee of the First Affiliated Hospital of Shandong First Medical University (Hospital Ethics Review No. S654). All the above data have been ethically reviewed.

### 2.2 Research variables

Based on clinical expertise and previous research evidence, including demographic characteristics (age, sex, smoking, and drinking status), physical measurements (BMI), medical history (hypertension and diabetes mellitus), and laboratory test results (white blood cell count, neutrophils, lymphocytes, and other relevant biomarkers). The neutrophil-to-lymphocyte ratio (NLR) was calculated by dividing the neutrophil count by the lymphocyte count (15).

### 2.3 Feature screening

Boruta is a random forest-based feature selection algorithm used to evaluate the importance of variables and identify features significantly associated with the target variable (16). We implemented this algorithm in R using the “Boruta” package, with parameters set to “pValue = 0.01” and “maxRuns = 500”. The algorithm generates random shadow variables and compares their importance distribution with that of the actual variables, iteratively assessing the significance of each variable. After a maximum of 500 iterations or when the variable importance stabilizes, the algorithm finalizes the selection results and identifies significant features.

LASSO regression applies a penalty function to shrink certain regression coefficients, imposing a constraint on the sum of their absolute values to keep it below a predetermined threshold, thereby enhancing the model’s robustness (17). We performed LASSO regression using the glmnet package in R, setting the family parameter to “binomial” to suit our binary outcome data. The key parameter, alpha, was set to 1, fully utilizing the LASSO method. Through cross-validation using the cv.glmnet function, we selected two lambda values: lambda.min, which minimizes the cross-validation error, and lambda.1se, which offers a more parsimonious model. These two values help strike a balance between model complexity and predictive accuracy. Finally, we filtered out the variables that contributed meaningfully to prediction based on non-zero coefficients, thereby simplifying the model and enhancing its interpretability.

As previously reported in the literature (16), we adopted the intersection of features identified by both Boruta and LASSO as the final set of predictors. This approach balances model complexity and interpretability while ensuring robust feature selection.

### 2.4 Algorithm development and validation

The predictive model was generated by partitioning the complete dataset into two mutually exclusive subsets. Seventy percent of the data was used for the training set, while the remaining 30% was allocated to the test set. The test set was reserved exclusively for final model evaluation and was not involved in any model training, feature selection, hyperparameter tuning, or validation procedures. All model development steps, including cross-validation and hyperparameter optimization, were conducted solely within the training set to prevent data leakage and ensure robust evaluation. Various machine learning algorithms were employed to develop the models, including logistic regression (LR), random forest (RF), support vector machine (SVM), decision tree (DT), k-nearest neighbors (KNN), Naive Bayes (NB), stochastic gradient boosting (SGBT), and neural network (NNET), as well as extreme gradient boosting (XGB). These machine learning algorithms were implemented using the Python package “Scikit-learn (version 0.24.1)”. These algorithms were selected for their ability to model complex relationships between variables and their robustness in handling both linear and nonlinear data structures. The training process employed 10-fold cross-validation, ensuring that the model was trained on different subsets of the data to improve its generalization capability and prevent overfitting. For each model, hyperparameter tuning was performed using grid search, evaluating a range of possible hyperparameters to identify the optimal value for each algorithm. To enhance the performance of the predictive models, the optimal hyperparameters for each model were identified through a combination of 10 rounds of 10-fold cross-validation and hyperparameter grid search, utilizing the best feature subset (**Supplementary Table S1**).

The performance of the machine learning models was evaluated using the testing set, which had not been involved in the training process. Key evaluation metrics, including accuracy, sensitivity, specificity, positive predictive value (PPV), negative predictive value (NPV), F1-score, kappa score, and area under the receiver operating characteristic curve (AUC), were calculated to assess the model’s ability to accurately classify individuals at risk for DPN. Additionally, decision curve analysis (DCA) was performed to evaluate the clinical utility of the models.

### 2.5 Model explanation

Explaining ML models can be challenging, especially with complex models often referred to as “black-box” models. The Shapley Additive Explanations (SHAP) method, based on game theory, provides a solution to this issue by ranking the importance of input features and explaining the results of predictive models. SHAP calculates the contribution of each feature to the prediction, offering both local and global explanations, thereby enhancing the transparency and interpretability of the model (9, 18). Interpretability analysis was performed using the SHAP Python library (version 0.43.0).

### 2.6 Network calculator

To support clinical implementation, the final prediction model was deployed via a Shiny-based web platform. By inputting the relevant clinical variables, the application generates an individualized probability of DPN in diabetic patients.

### 2.7 Statistical analysis

The preliminary analysis of the dataset involved the application of descriptive statistics. In clinical data, continuous variables were expressed as mean ± standard deviation (SD), while categorical variables were described using frequencies and percentages. Statistical tests, such as the chi-square test and unpaired t-test, were used to compare variables between groups. All statistical analyses were performed using the R version 4.4.2 software package. A two-sided P value of < 0.05 was considered statistically significant.

## 3 Result

### 3.1 Patient characteristics

**Table 1** presents the characteristics of the cohort. This study included 1,544 diabetic patients (mean age: 64.39 years; 53% male). Baseline characteristics were compared between patients with DPN and patients without DPN, significant statistical differences were found between the groups in terms of age, gender, smoking status, hypertension, NE, LYM, NLR, Hb, uric acid, HbA1c, TC, SCR, Albumin, LDH, and diabetes duration (P < 0.05). The type 2 diabetes patients were randomly divided into the training group (n = 1,082) and the validation group (n = 462). In both groups, approximately 34.3% and 34.2% of the patients were diagnosed with DPN, respectively. The baseline characteristics of the two groups were similar (**Table 1**).

**Table 1:** Comparison of demographic characteristics and clinical characteristics between diabetic peripheral neuropathy (DPN) and non-DPN patients, and between training and test sets.

Characteristic	Total, N = 1,544	Non-DPN, N = 1,015	DPN, N =529	*P^1^*	Training set, N =1,082	Test set, N = 462	*P^1^*
PN, %							>0.99
Non-DPN	1,015 (65.7)				711 (65.7)	304 (65.8)	
DPN	529 (34.3)				371 (34.3)	158 (34.2)	
**Age, years**	64.39 (11.73)	62.77 (11.59)	67.50 (11.38)	<0.001	64.25 (11.63)	64.72 (11.95)	0.47
**Sex, %**				<0.001			0.47
Male	819 (53.0)	494 (48.7)	325 (61.4)		567 (52.4)	252 (54.5)	
Female	725 (47.0)	521 (51.3)	204 (38.6)		515 (47.6)	210 (45.5)	
BMI, %				0.99			0.97
Normal	252 (16.3)	165 (16.3)	87 (16.4)		175 (16.2)	77 (16.7)	
Overweight	554 (35.9)	365 (36.0)	189 (35.7)		388 (35.9)	166 (35.9)	
Obesity	738 (47.8)	485 (47.8)	253 (47.8)		519 (48.0)	219 (47.4)	
Smoking status, %				0.04			0.54
Now	352 (22.8)	242 (23.8)	110 (20.8)		245 (22.6)	107 (23.2)	
Former	89 (5.8)	67 (6.6)	22 (4.2)		58 (5.4)	31 (6.7)	
Never	1,103 (71.4)	706 (69.6)	397 (75.0)		779 (72.0)	324 (70.1)	
Hypertension, %				0.01			0.92
Yes	988 (64.0)	627 (61.8)	361 (68.2)		691 (63.9)	297 (64.3)	
No	556 (36.0)	388 (38.2)	168 (31.8)		391 (36.1)	165 (35.7)	
**WBC, × 10^9^/L**	7.46 (2.12)	7.42 (2.08)	7.53 (2.21)	0.34	7.68 (1.80)	7.71 (1.87)	0.72
**NE, × 10^9^/L**	4.46 (1.65)	4.38 (1.55)	4.61 (1.81)	0.001	4.45 (1.64)	4.47 (1.66)	0.82
**LYM, × 10^9^/L**	2.17 (0.88)	2.22 (0.88)	2.07 (0.86)	<0.001	2.18 (0.88)	2.14 (0.87)	0.48
**NLR**	2.35 (1.43)	2.24 (1.31)	2.58 (1.62)	<0.001	2.36 (1.51)	2.34 (1.23)	0.78
**Hb, g/L**	14.13 (1.59)	14.19 (1.56)	14.01 (1.66)	0.03	14.11 (1.59)	14.15 (1.61)	0.66
**Uric acid, µmol/L**	335.00 (92.21)	330.66 (91.81)	343.33 (92.50)	0.01	334.81 (93.96)	335.45 (88.09)	0.90
**HbA1c, %**	7.69 (1.82)	7.42 (1.81)	8.20 (1.72)	<0.001	7.68 (1.80)	7.71 (1.87)	0.72
**ALT, U/L**	26.56 (19.54)	26.88 (19.01)	25.95 (20.51)	0.38	26.44 (19.86)	26.82 (18.78)	0.73
**AST, U/L**	25.44 (15.75)	25.47 (16.05)	25.39 (15.17)	0.93	25.45 (16.23)	25.43 (14.58)	0.99
**TBil, mmol/L**	11.28 (4.74)	11.28 (4.69)	11.26 (4.83)	0.94	11.27 (4.87)	11.30 (4.42)	0.90
**TC, mmol/L**	5.24 (1.21)	5.32 (1.23)	5.10 (1.17)	0.001	5.27 (1.20)	5.19 (1.25)	0.23
**TG, mmol/L**	2.34 (2.58)	2.36 (2.68)	2.31 (2.40)	0.73	2.36 (2.49)	2.30 (2.79)	0.66
**SCR, μmol/L**	88.34 (70.03)	78.99 (37.05)	106.28 (105.85)	<0.001	88.33 (69.44)	88.36 (71.45)	>0.99
**Albumin, g/dl**	41.74 (3.44)	41.94 (3.37)	41.36 (3.56)	0.002	41.66 (3.46)	41.93 (3.40)	0.17
**Globulin, g/dl**	32.01 (5.03)	31.92 (4.82)	32.16 (5.41)	0.37	32.03 (4.95)	31.95 (5.21)	0.76
**LDH, U/L**	143.70 (35.69)	140.31 (33.51)	150.22 (38.75)	<0.001	143.12 (35.28)	145.07 (36.63)	0.33
**ALP, U/L**	81.59 (30.58)	80.78 (31.11)	83.14 (29.50)	0.15	82.02 (31.67)	80.58 (27.87)	0.40
**Diabetes duration, years**	7.08 (12.41)	6.24 (11.45)	8.69 (13.94)	<0.001	6.86 (12.07)	7.60 (13.16)	0.29

PN, peripheral neuropathy; BMI, body mass index; WBC, white blood cell; NE, neutrophil; LYM, lymphocyte; NLR, NE-to-LYM ratio; Hb, hemoglobin; ALT, glutamic pyruvic transaminase; AST, glutamic oxaloacetic transaminase; TBil, total bilirubin; TC, total cholesterol; TG, thyroglobulin; SCR, serum creatinine; LDH, lactate dehydrogenase; ALP, alkaline phosphatase. 1Pearson’s Chi-squared test; Wilcoxon rank sum test.

### 3.2 Predictor screening

**Figure 2:**
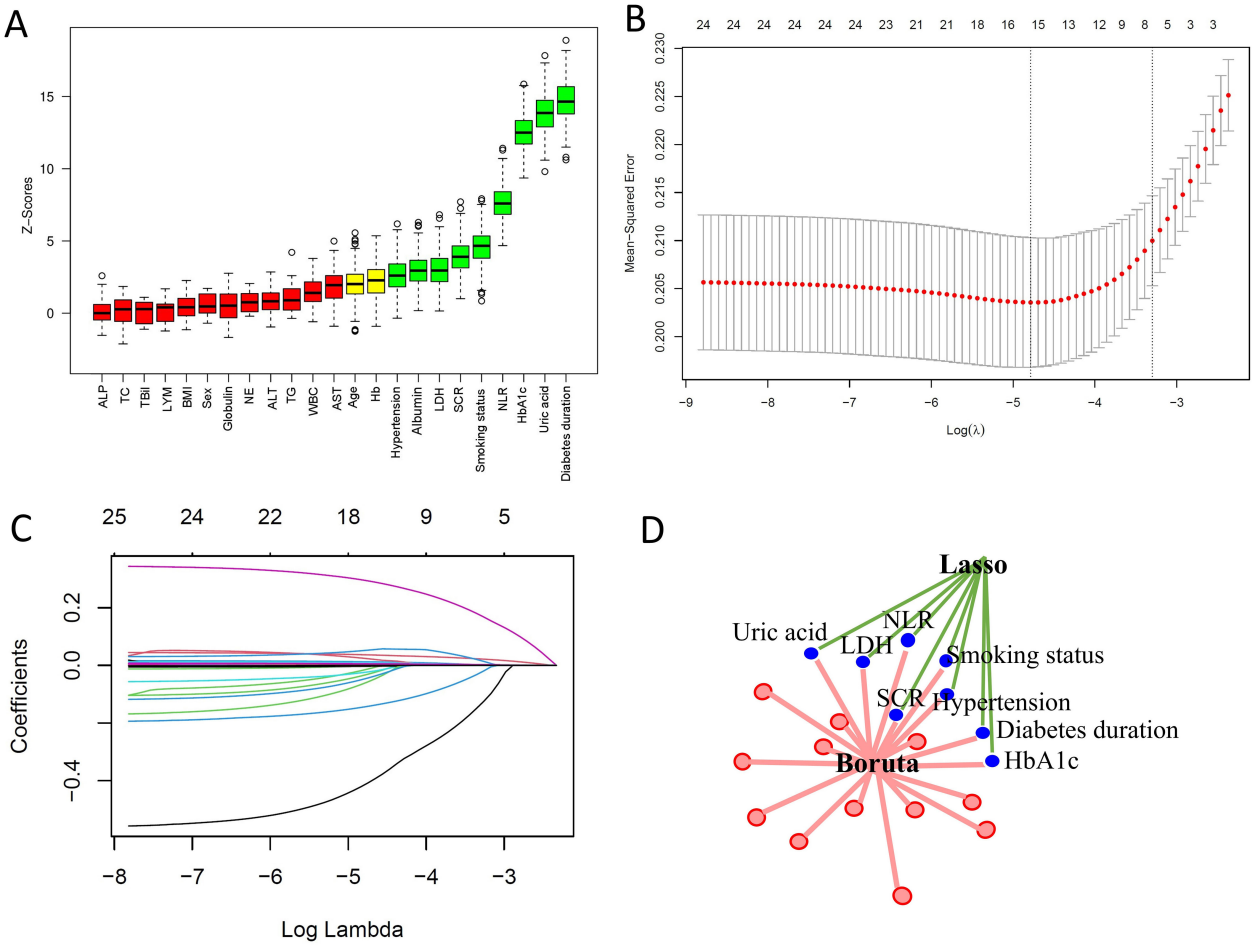
Predictor screening results. **(A)** Boruta feature selection. **(B)** LASSO screening with lambda values indicated by dashed lines. **(C)** Variable trajectories in the LASSO model. **(D)** Common predictors identified by Boruta and LASSO, including diabetes duration, HbA1c, NLR, SCR, hypertension, smoking status, LDH, and uric acid.

The Boruta algorithm is an extension of the random forest method that accurately estimates the importance of each feature to identify the actual feature set. The Boruta algorithm identified 9 key factors, including diabetes duration, uric acid, HbA1c, NLR, smoking status, SCR, LDH, Albumin, and hypertension (**Figure 2A**). In contrast, LASSO regression is a shrinkage estimation method that performs variable selection and complexity adjustment by formulating an optimization objective function that includes a penalty term. In this study, LASSO regression was used to identify features such as diabetes duration, uric acid, HbA1c, NLR, smoking status, SCR, LDH, and hypertension (**Figures 2B, C**). By comparing the results obtained from the Boruta algorithm and LASSO regression, we identified the common subset of features selected by both methods. These selected features were ultimately used to construct the model, including diabetes duration, uric acid, HbA1c, NLR, smoking status, SCR, LDH, and hypertension (**Figure 2D**).

### 3.3 Model performance

**Figure 3:**
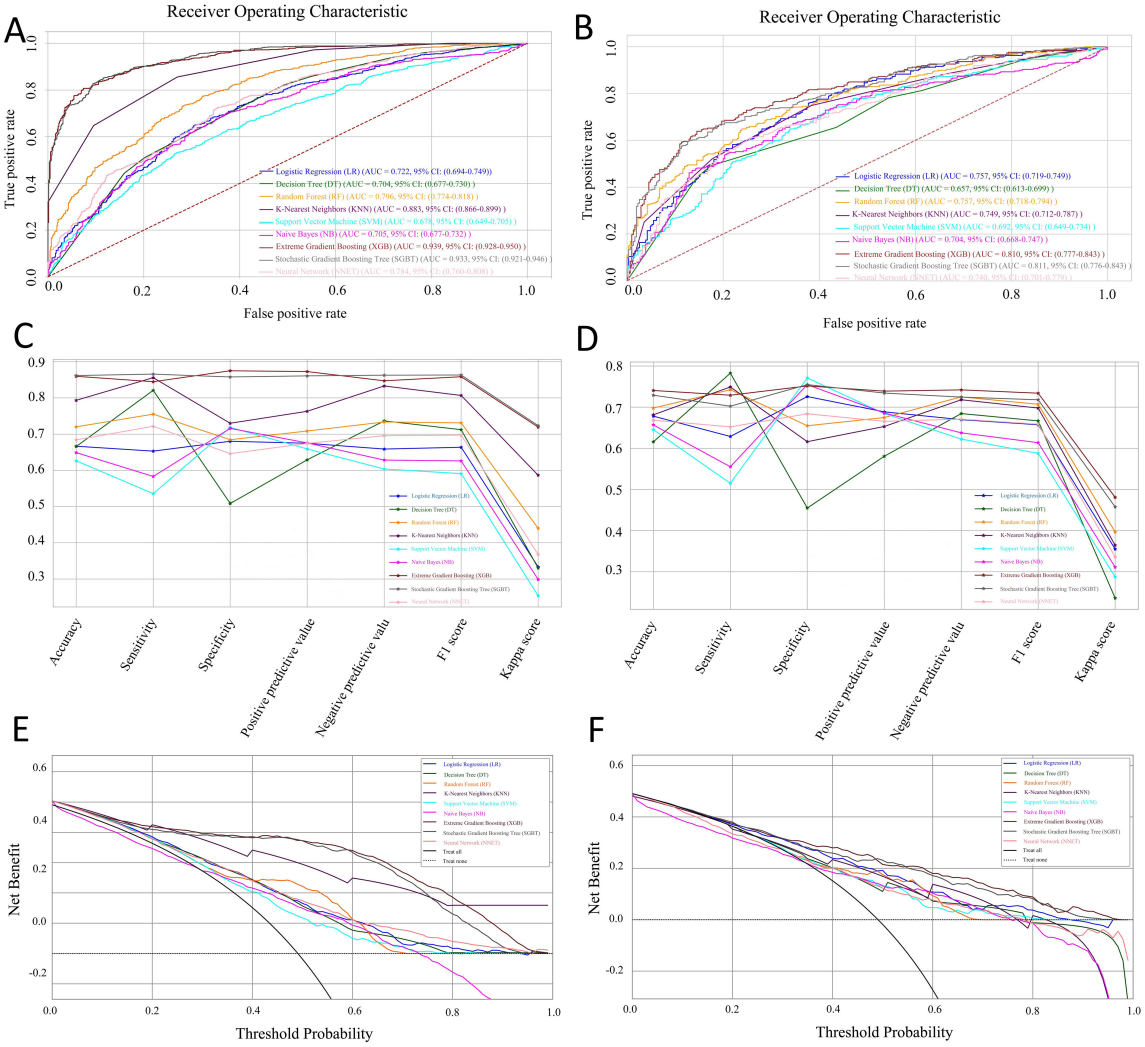
Performance and comparison of nine predictive models. ROC curves for the training set **(A)** and the test set **(B)**. Evaluation metrics for the training set **(C)** and the test set **(D)**, including accuracy, sensitivity, specificity, PPV, NPV, F1 score, and kappa value. Decision Curve Analysis (DCA) for the training set **(E)** and the test set **(F)**.

We performed ten rounds of 10-fold internal cross-validation and developed nine machine learning models. In the training dataset, the AUC values for the models were as follows: LR 0.722 (95% CI: 0.694-0.749), DT 0.704 (95% CI: 0.677-0.730), RF 0.796 (95% CI: 0.774-0.818), KNN 0.883 (95% CI: 0.866-0.899), SVM 0.678 (95% CI: 0.649-0.705), NB 0.705 (95% CI: 0.677-0.732), XGB 0.939 (95% CI: 0.928-0.950), SGBT 0.933 (95% CI: 0.921-0.946), and NNET 0.784 (95% CI: 0.760-0.808) (**Figure 3A**). In the test set, the SGBT model demonstrated superior predictive performance with an AUC of 0.811 (95% CI: 0.776-0.843). In comparison, the AUC values for the remaining models in the test set were as follows: LR 0.757 (95% CI: 0.719-0.749), DT 0.657 (95% CI: 0.613-0.699), RF 0.757 (95% CI: 0.718-0.794), KNN 0.749 (95% CI: 0.712-0.787), SVM 0.692 (95% CI: 0.649-0.734), NB 0.704 (95% CI: 0.668-0.747), XGB 0.810 (95% CI: 0.777-0.843), and NNET 0.740 (95% CI: 0.701-0.779) (**Figure 3B**). The accuracy, sensitivity, specificity, PPV, NPV, F1 score, and kappa values were calculated and compared for models within the training set (**Figure 3C**) and the test set (**Figure 3D**). The DCA demonstrated that, in the training set, the SGBT model outperformed all other models across the entire threshold range (0–0.8), followed by the XGB model (**Figure 3E**). In the test set, the RF model provided the best performance across the full threshold range (0–1.0) (**Figure 3F**).

### 3.4 Model explanation

**Figure 4:**
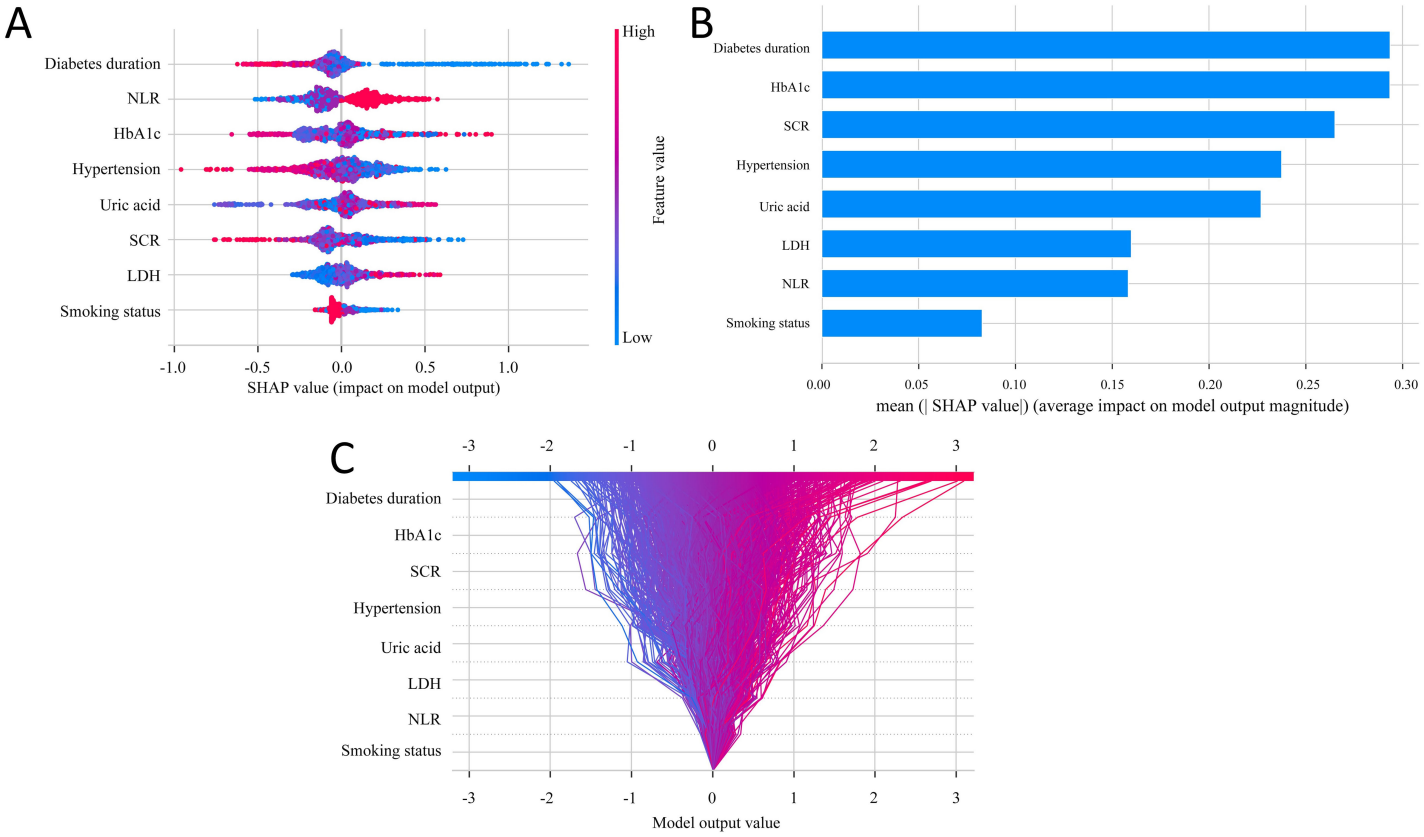
SHAP analysis for feature interpretability. **(A)** SHAP dendrogram of features for the SGBT model. **(B)** Feature importance ranking for the logistic regression model. **(C)** Decision plot of feature contributions to the model outputs.

We employed the SHAP method to interpret the final model’s output by calculating the contribution of each variable to the prediction. In **Figure 4A**, feature importance is visualized, where each point corresponds to a sample, and a color gradient from blue (low values) to red (high values) reflects the magnitude of the feature value. The vertical axis displays the ranked features, showing the correlation and distribution of feature values with their corresponding SHAP values. **Figure 4B** presents the average SHAP values for each feature, ordered by importance in descending order on the vertical axis. The analysis reveals that diabetes duration, HbA1c, SCR, hypertension, and uric acid are the top five most influential features, indicating their critical role in predicting DPN. **Figure 4C** provides a decision plot, which illustrates the contribution of each feature to the final prediction. This plot also tracks the changes in SHAP values for individual samples, offering insights into feature interactions and the model’s decision-making process.

### 3.5 Implementation of the web calculator

**Figure 5:**
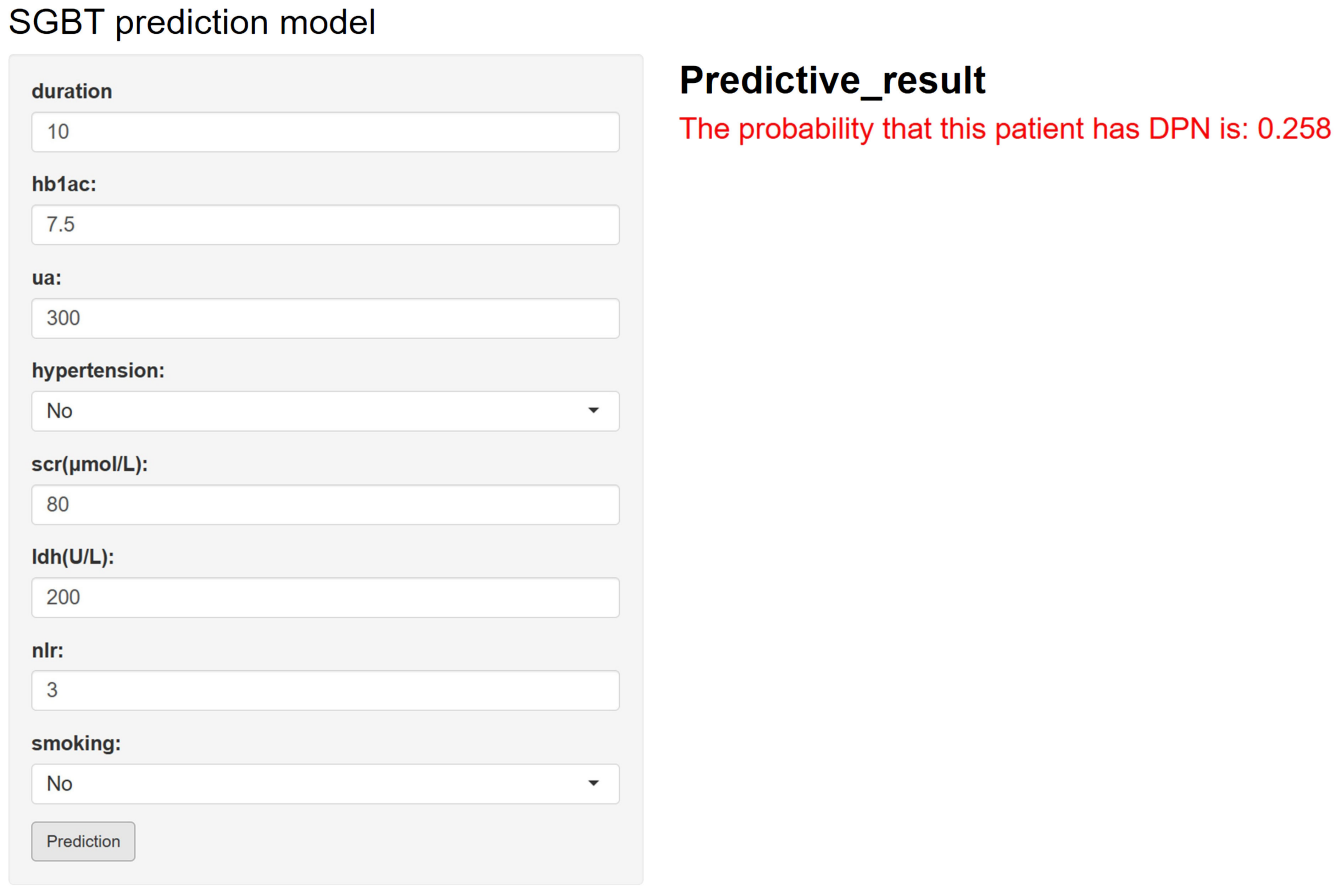
Web-based calculator for predicting the risk of diabetic peripheral neuropathy (DPN) in patients with diabetes using the developed model. By entering values for diabetes duration, uric acid, HbA1c, NLR, smoking status, serum creatinine (SCR), lactate dehydrogenase (LDH), and hypertension, an individualized DPN risk prediction can be obtained.

As illustrated in **Figure 5**, the final SGBT model was deployed as an interactive web application to facilitate clinical use. By entering the values of the eight selected features, clinicians can obtain an individualized risk estimate for DPN. The tool is available online at: https://dpn-prediction.shinyapps.io/shiny-sgbt/.

## 4 Discussion

DPN is one of the most common complications in patients with T2DM, characterized by its progressive, irreversible, and debilitating nature (19, 20). With the increasing prevalence of diabetes, DPN has become a significant global public health issue. DPN not only severely impacts the quality of life of patients but also leads to long-term medical costs and substantial economic burden. Without timely diagnosis and intervention, DPN can result in severe complications such as lower limb ulcers, infections, and even amputations, all of which significantly increase mortality and disability rates. Therefore, developing effective early screening and risk assessment tools is of crucial importance for the early diagnosis, intervention, and prevention of DPN. This study aims to assess and predict the risk of DPN using ML techniques. We compared nine different machine learning models for analyzing and predicting the risk of DPN, with the goal of developing a predictive tool that can effectively identify the risk of DPN.

This study employed a dual approach combining the Boruta algorithm and LASSO regression to ensure accurate feature selection and model stability. The final predictive factors identified included diabetes duration, uric acid, HbA1c, NLR, smoking status, SCR, LDH, and hypertension. Most of these factors have all been previously shown to be closely associated with the development of DPN. Numerous studies indicate that patients with a longer duration of T2DM are more likely to develop DPN (21, 22). Persistent hyperglycemia has been found to induce the formation of advanced glycation end products (AGEs), which bind to proteins in nerve cells and vascular endothelial cells, causing cellular damage, vascular injury, and neural dysfunction (23, 24). Over time, the accumulation of AGEs may lead to chronic inflammation and oxidative stress in neural tissues, further damaging peripheral nerves. HbA1c is widely recognized as the optimal biochemical marker for assessing long-term metabolic control in diabetes patients. Numerous prospective studies have confirmed a strong association between HbA1c and diabetic complications, a conclusion consistent with our findings (25–27). Our study also confirmed that uric acid and SCR are significant risk factors for DPN. Since peripheral nerves and renal vasculature are both exposed to the diabetic environment, it is generally believed that the development and progression of diabetic nephropathy (DN) and DPN occur concurrently (28, 29). A model was developed to predict DN, analyzing the risk factors for microvascular complications in T2DM patients, and it was found that DPN is closely related to DN (30). Further studies observed a significant correlation between different stages of DN and neuropathy in type 2 diabetes patients (31).

Additionally, our findings demonstrate that NLR is an important risk factor for DPN, consistent with previous studies (32). High NLR values are often strongly associated with endothelial dysfunction (33), which can lead to insufficient blood supply to peripheral nerves. Enzymes and reactive oxygen species released by neutrophils can damage vascular endothelial cells, exacerbating microcirculatory disorders (34). Moreover, elevated NLR levels reflect increased levels of pro-inflammatory cytokines, such as TNF-α and IL-1β. These cytokines exhibit direct neurotoxicity, accelerating neuronal apoptosis and functional loss (35).

In this study, we developed and compared the performance of nine ML models, including LR, RF, SVM, DT, KNN, NB, SGBT, NNET, and XGB. Among these, XGB and SGBT demonstrated the highest discriminative ability on the training set, with AUCs of 0.939 (95% CI: 0.928–0.950) and 0.933 (95% CI: 0.921–0.946), respectively. On the test set, the AUC of SGBT was 0.811 (95% CI: 0.776–0.843), slightly outperforming XGB at 0.810 (95% CI: 0.777–0.843). Although the performance difference between these models on the test set was marginal, SGBT exhibited greater stability and generalization capability, making it the optimal choice for this study. The superior generalization performance of SGBT is particularly valuable in ensuring robustness and reliability when applied to real-world scenarios, where data distributions can be complex and variable.

Although the SGBT model demonstrated the best performance among the nine ML models, the performance gap between the training set (AUC: 0.933) and the testing set (AUC: 0.811) suggests a potential risk of overfitting. This discrepancy may be attributed to model complexity, limited sample size, or inherent data heterogeneity. In light of the data imbalance in this study, one potential approach to mitigate this overfitting could be applying techniques such as SMOTE to balance the dataset. SMOTE has been shown to improve model generalization by synthesizing new samples from the minority class, which could lead to a more balanced training process and potentially reduce overfitting. However, we did not apply SMOTE in this study to avoid altering the real-world distribution of DPN cases, but it remains a strategy for future exploration. Additionally, expanding the sample size and incorporating external validation cohorts would be beneficial in further assessing the model’s robustness.

To further enhance the interpretability of the selected model, we utilized SHAP, a method designed to address the “black-box” nature of ML algorithms (36, 37). SHAP effectively elucidated the contributions of individual features, highlighting key clinical indicators such as diabetes duration, HbA1c, SCR, hypertension, and uric acid. These features, widely recognized as critical factors in the development of DPN, were shown to play a significant role in the ML framework used for risk prediction. Among them, longer diabetes duration and elevated HbA1c levels emerged as the most influential predictors. These findings are consistent with previous studies (38–40), which have demonstrated that chronic hyperglycemia and prolonged disease duration contribute to the development of diabetic neuropathy by promoting oxidative stress, inflammation, and microvascular damage. This alignment with clinical evidence enhances the model’s interpretability and supports its clinical applicability.

Compared with existing DPN prediction models, our SGBT model demonstrated competitive performance. For example, one prior study utilizing logistic regression reported an AUC of 0.759 (41), whereas our model achieved a higher AUC of 0.811 on the independent test set, indicating better predictive capability and generalizability. On the other hand, a recent study by Jiang et al. (42) reported a higher AUC of 0.900. Their model integrated both conventional clinical variables and unique indicators derived from traditional Chinese medicine, potentially capturing more comprehensive biological signals and improving overall predictive accuracy. In contrast, our model was developed solely using routine clinical and biochemical variables, which enhances its practicality, reproducibility, and ease of implementation in standard healthcare settings. Furthermore, our model features SHAP-based interpretability and a web-based risk calculator to facilitate clinical translation.

Despite the achievements of this study, there are some limitations to acknowledge. First, as a retrospective study, issues such as missing data and selection bias may affect the reliability of the results. Second​​, the cohort primarily consisted of urban patients receiving standardized treatment protocols, which may not fully represent rural populations or institutions with varying healthcare delivery practices. Third, the relatively small sample size and lack of adjustment for key covariates, such as lifestyle factors and medication regimen, may limit the generalizability of the findings to the broader population. To address these limitations, future research should adopt a prospective, multicenter design with larger and more diverse cohorts, ensuring enhanced representativeness and external validity. Additionally, exploring novel biomarkers and dynamic risk factors, along with incorporating real-time monitoring data, may enrich the model’s predictive capabilities. Such advancements would facilitate its clinical application, ultimately enabling more precise diagnosis and personalized management strategies for DPN patients.

## 5 Conclusion

This study developed an effective predictive tool for DPN using the SGBT model. By identifying and analyzing key predictive factors, it establishes a solid scientific foundation for the early detection and prevention of DPN, aiming to reduce complications and improve patients’ quality of life. Future efforts should focus on further validating the model’s performance, enhancing its accuracy and practical applicability, and integrating it into clinical workflows to advance personalized management and improve outcomes for DPN patients.

## Data Availability

The original contributions presented in the study are included in the article/**Supplementary Material**. Further inquiries can be directed to the corresponding authors.
