# Supplementary material for: Machine learning-based prediction of diabetic peripheral neuropathy: model development and clinical validation
Source: Front Endocrinol (Lausanne). 2025 Jun 5;16:1614657. doi: 10.3389/fendo.2025.1614657 (PMC12176554; doi:10.3389/fendo.2025.1614657)
Supplement: Supplementary file 1 [file Table1.docx]

Supplementary Material

**Table S1. Hyperparameters for nine models**

| Model | Hyperparameter | Candidate Hyperparameter Ranges |
| --- | --- | --- |
| Logistic Regression (LR) | none | none |
| Decision Tree (DT) | cp = 0.053 | cp: [0.01, 0.1] |
| Random Forest (RF) | mtry =4, min.node.size=1, splitrule="gini" | mtry: [1, 5], min.node.size: [1,10], splitrule: ["gini","entropy"] |
| Support Vector Machine (SVM) | sigma = 0.008521, C = 0.1823 | sigma: [10⁻³,10³] (log), C: [10⁻³,10³] (log) |
| K-Nearest Neighbors (KNN) | k = 4 | k: [1,20] (integers) |
| Naive Bayes (NB) | usekernel = FALSE, laplace = 0, adjust =1 | useKernel: [TRUE, FALSE], adjust: [0.5,2], |
| Extreme Gradient Boosting (Xgboost) | eta = 0.2, max_depth = 9, gamma =0.1, colsample_bytree= 0.8, min_child_weight= 0.9, subsample=0.5, nrounds=100 | eta: [0.01,0.3], max_depth: [3,15], gamma: [0,5], colsample_bytree: [0.5,1], min_child_weight: [0.5,2], subsample: [0.5,1], nrounds: [50,200] (integers) |
| Stochastic Gradient Boosting Tree (SGBT) | shrinkage = 0.3, interaction.depth = 9, n.minobsinnode =10, n.trees= 30 | shrinkage: [0.01,0.5], interaction.depth: [1,10], n.minobsinnode: [5,20], n.trees: [20,100] |
| Neural Network (NNET) | size = 2, decay = 0.01 | size: [1,10], decay: [10⁻⁴,0.1] (log) |
